# Zygomatic implants placed in atrophic maxilla: an overview of current systematic reviews and meta-analysis

**DOI:** 10.1186/s40902-020-00286-z

**Published:** 2021-01-06

**Authors:** Shaqayeq Ramezanzade, Julian Yates, Frank J. Tuminelli, Seied Omid Keyhan, Parisa Yousefi, Jose Lopez-Lopez

**Affiliations:** 1 School of Dentistry, Isfahan University of Medical Science, Isfahan, Iran; 2 Department of Oral and Maxillofacial Surgery, University of Manchester, Manchester, UK; 3 Department of Dental Medicine, Hofstra Northwell School of Medicine, New York, USA; 4 Maxillofacial Surgery and Implantology Research Foundation., Tehran, Iran; 5 Craniomaxillofacial Research Center for Craniofacial Reconstruction, Tehran University of Medical Science, Shariati Hospital, Tehran, Iran; 6 Department of Prosthodontics, Dental College, Isfahan University of Medical Science, Isfahan, Iran; 7 Department of Odontostomatology, School of Dentistry & Dental Hospital Barcelona University, University of Barcelona, Feixa Llarga, s/n – L’Hospitalet de Llobregat, 08907 Barcelona, Spain

**Keywords:** Umbrella review, Zygomatic implant, Atrophic, Zygomatic

## Abstract

**Background:**

Zygomatic implants are a treatment option for severely atrophic maxilla.

**Main text:**

This study aimed to summarize and evaluate systematic reviews assessing the clinical outcomes of zygomatic implants including survival/failure rate and complications. PubMed-MEDLINE, Google Scholar, LILACS, and the Cochrane Database were searched up to April 2020. Risk of bias assessment was conducted by the AMSTAR tool. Initial searches yielded 175 studies. These were assessed, and following title abstract and full-text evaluation, 7 studies (2 meta-analyses) were included in the final review. According to the AMSTAR tool, 1 was deemed high quality, 4 were classified as medium, and 2 as low quality. The mean AMSTAR score (±SD) was 5.28 of 9 (±2.36) ranging from 2/9 to 9/9. The reported survival rates ranged from 95.2 to 100% except for resected maxillas, which established higher failure rates up to 21.43%. Concerning the complications with the zygomatic implants, various surgical and prosthetic complications were reported with sinusitis being the most frequently observed complication. Zygomatic implants appears to offer a promising alternative to formal bone grafting techniques with lower costs, less complications, less morbidity, shorter treatment times, and comparably high survival rates.

**Conclusion:**

Complications were rare and usually easy to manage. However, the treatment should be directed by appropriately trained clinicians with noticeable surgical experience.

## Introduction

In patients with sufficient bone volume in the edentulous or semi-dentate maxilla, rehabilitation of masticatory function with dental implants can be achieved with predictable success and acceptable long-term results. However, due to mechanical and anatomical difficulties, rehabilitation of severely resorbed maxilla rehabilitation with endosseous implants remains a challenge [1, 2]. Several surgical procedures have been advocated to treat the atrophic maxilla including grafting techniques (block, composite, Le Fort I inter-positional and iliac crest, and maxillary sinus grafts), sinus floor elevation, and guided bone regeneration (GBR) [3–5]. There are also less aggressive alternatives including short implants, tilted implants, and zygomatic implants [6, 7].

To ensure acceptable success rates for standard dental implants without any bone augmentation procedures, the minimal bone height in the posterior region of the maxilla needs to be at least 10 mm [8]. Although in the posterior atrophic maxilla where the height of residual bone is at least 6–7 mm, and where the width of any residual ridge permits placement of at least 5-mm-diameter implants, short implants can be a safe choice [9]. Nevertheless, there are reports that short implants, alone or in conjunction with sinus floor elevation, less than 6 mm significantly diminished implant survival rate [3].

In cases where residual bone height and width does not permit placement of conventional dental implants, surgical procedures as well as ZIs (abbreviation of zygomatic implants) can be considered.

Over the past decades, different bone grafting procedures have been advocated prior to, or simultaneously with, implant placement in routine implant treatments with the aim of increasing the volume of load-bearing bone [10]. Conventional grafting with autogenous bone has been considered the “gold standard” in the treatment of the extremely atrophic maxillae, but due to high failure rates of 10–30%, additional time and higher costs, the development and introduction of a new standard with superior clinical outcomes is warranted [11].

Based on experiences in human and animal studies, Branemark et al. [12] reported that the introduction of an implant in the maxillary sinus would not necessarily jeopardize sinus health. Likewise, by considering high success rates using the zygomatic bone as an anchorage point for prosthetic rehabilitation in patients with maxillary defects [13], they developed a new type of implant named the zygomaticus fixture, which could obtain implant anchorage and stability in the zygoma. ZIs as described by Malevez et al. [14] are machined surface, self-tapping screws in commercially pure titanium which present a 45° angulated prosthetic head to compensate for the angulation between the zygomatic bone and the alveolus. By increasing the implant length with ranges from 30 to 52.5 mm, ZI could be placed in the atrophic upper jaws even with poor bone quality.

When considering all the available surgical options, treatment choice will be dependent on the characteristics of the patient, the amount of residual bone, and the general risks and the patient’s wishes to undertake [5, 15]. In severe maxillary atrophy, ZI offers a viable alternative to the rather invasive procedures including bone grafting or sinus-lift procedures since they eliminate the necessity of onlay bone grafting or sinus augmentation (and thus a graft donor site); a smaller number of implants are necessary to support fixed prostheses. Furthermore, in many instances, formal grafting is not a viable alternative due to the residual morphology of the atrophic maxillae following resorption. ZIs may also decrease patient morbidity, treatment time, and costs [15, 16].

To date, several systematic reviews (SR) have tried to elucidate the weight of evidence of clinical outcomes when utilizing ZIs, including survival/failure rate and complications. However, to the best of our knowledge, this is the first overview evaluating all evidence from the existing SR on this topic using an umbrella approach.

## Methods and materials

### Protocol and registration

The reporting of this study was based on the PRISMA checklist [17]. The clinical questions were identified using a PICOS (population, intervention, comparisons, outcomes, study design) strategy, and the search protocol was specified and made publicly available at PROSPERO (http://www.crd.york.ac.uk/PROSPERO). The registration number is CRD42020179991.

*PICO* entails the following: Patient: systematic reviews and meta-analysis on ZIs placed in atrophic maxilla; Intervention: ZIs placed in upper atrophic jaw; Comparison: other treatment choices for implant prosthetic treatment of atrophic maxilla or no comparison; Outcome: implant survival/failure, complications, bone level variation (mm).

### Inclusion criteria

Systematic reviews with/without meta-analyses dealing with ZIs treating atrophic maxilla were considered eligible for inclusion. We considered reviews as “systematic review” if they matched the following description, as proposed by the Cochrane Collaboration’s Handbook (Chapter 1.2.2) [18]: “It uses explicit, systematic methods that are selected with a view to minimizing bias, thus providing more reliable findings from which conclusions can be drawn and decisions made.” There was no limitation for the year of publication. We included articles published in English.

The outcome variables were the following:

A. Implant survival/failure
B. Complications (surgical/prosthetic)

Details of the included studies can be seen in Table 2.

### Exclusion criteria

Primary or original clinical research, abstracts, animal studies, in vitro studies, case reports, case series, letters to the editor and narratives, conference papers, other types of non-systematic reviews (e.g., critical reviews, overviews, state-of-the-art reviews), and revisions of in vitro or animal studies were excluded. Also systematic reviews/meta-analysis with less than 3 included articles will be excluded. The reasons for excluding articles are also recorded in Table 1.

**Table 1:** List of excluded articles after reading full-text and the reason for exclusion

Study (first author/year)	Reason for exclusion
Candel-marti et al. [6]	Not a systematic review (review of literature)
Sorni et al. [19]	Not a systematic review (review of literature)
Vega et al. [20]	Not a systematic review (review of literature)
Filho et al. [21]	Not a systematic review (review of literature)
Neugarten et al. [22]	Not a systematic review (a retrospective chart review)
Hackett et al. [23]	Not a systematic review (review of literature)
Aparicio et al. [11]	Not a systematic review (review of literature)
Bedrossian et al. [24]	Not a systematic review (a review and clinical experiences)
Cid cisternas et al. [25]	Not a systematic review (review of literature)
Davo et al. [26]	Not a systematic review (review of literature)
Dominguez et al. [27]	Not a systematic review (review of literature)
Block et al. [28]	Not a systematic review (review of literature)
Galan et al. [29]	Not a systematic review (review of literature)
Malevez et al. [14]	Not a systematic review (review of literature)
Meenakshi et al. [30]	Not a systematic review (review of literature)
Pandita et al. [31]	Not a systematic review (review of literature)
Prithviraj [32]	Not a systematic review (review of literature)
Rosenstein et al. [33]	Not a systematic review (review of literature)
Gulia et al. [34]	Not a systematic review (review of literature)
Arean et al. [35]	Not a systematic review (review of literature)
Sorni et al. [19]	Not a systematic review (review of literature)
Chrcanovic et al. [36]	Not a systematic review (review of literature)
Malevez et al. [37]	Not a systematic review and not English
Pineau et al. [38]	Not a systematic review and not English
Esposito et al. [39]	Included reviews less than 3 studies
Esposito et al. [40]	Included reviews less than 3 studies
Jokstad et al. [7]	ZI variables were not reported as a primary outcome
Sharma et al. [16]	ZI variables were not reported as a primary outcome
Galve et al. [41]	Conference paper
Garcia et al. [42]	Conference paper

### Information sources and search strategy

Using mesh terms and other keywords relating to this topic, we searched the following electronic databases (up to April 2020): PubMed-Medline, Google Scholar, and the Cochrane Database of Systematic Reviews, LILACS (Latin American and Caribbean Health Sciences Literature), and reference lists of included SR were searched manually to identify the potential pertinent papers. The following terms were used to conduct searching:

Search terms included the following:

PUBMED> [(zygomatic) OR (zygoma) OR (zygomaticus)] AND [(implant) OR (implants) OR (fixture) OR (fixtures)] AND [(Review) OR (systematic review) OR (meta-analysis)]

Google Scholar> allintitle: review “zygomatic” OR “zygoma zygomaticus” OR “zygomatic implant” OR “zygomatic implants” OR “zygomatic fixture” OR “zygomatic fixtures”

This search strategy was adapted to other searched electronic databases. The computer software, EndNote X8, Thomson Reuters, was used to manage references.

### Study selection

The search for eligible studies was conducted by 2 reviewers in an independent manner to determine proper SR using the inclusion and exclusion criteria. To select potentially eligible studies, the two reviewers read titles and/or abstracts. If appropriate, then full-text articles were assessed for validity. Any disagreement between reviewers was resolved involving a third operator.

### Data extraction

The following data was collected from the articles by an author (SHR) based on a predefined checklist worksheet and supervised by the other authors for accuracy (JLL, FT, and JY):

Summary of SR characteristics: Authors’ name and year of publication, country of origin, journals’ impact factor, source of funding, conflict of interest, number and type of studies included, number of implants, presence of meta-analysis (yes/no), data bases searched, search date, follow-up, quality assessment tool, main outcome(s), main conclusion(s), study-specific relative risk estimates (risk ratio, odds ratio, or standardized mean differences) along with the corresponding 95% confidence intervals (CI), *P* value.

### Risk of bias assessment in individual studies

The risk of bias assessment was performed by two reviewers independently (SHR and JLL) and supervised by JY and FT using the AMSTAR tool [43]. For each systematic review, 11 items of AMSTAR [43] were answered using “yes,” “no,” or in some cases “can’t answer.”

## Results

**Fig. 1:**
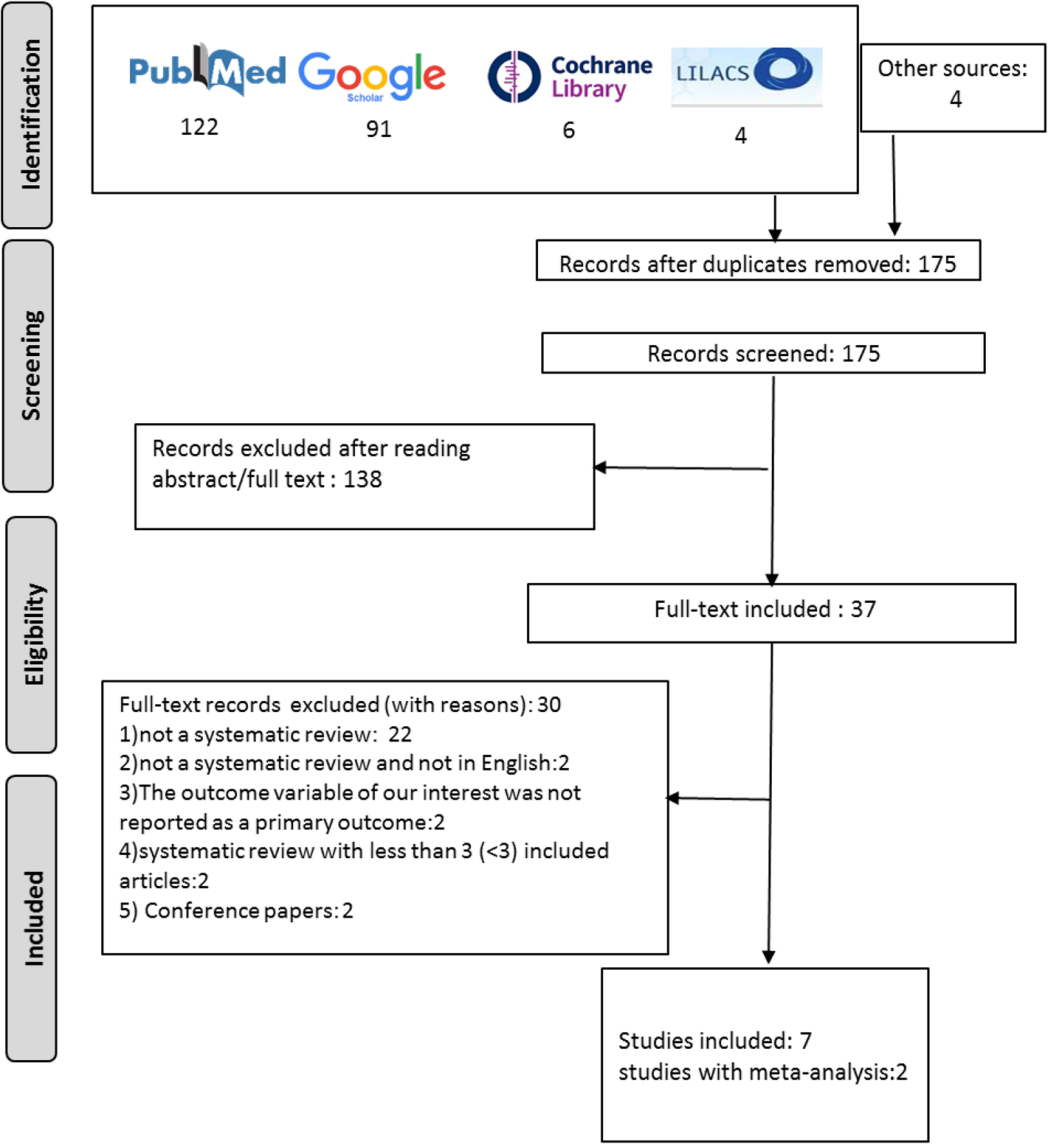
Flow diagram of included SRs according to PRISMA guideline [17]

The flow diagram of the study selection process is shown in Fig. 1. A total of 223 references were obtained through searching the databases and additional 4 studies were retrieved by manual search. One hundred seventy five studies were obtained after duplication removal. Of these, 138 were excluded by reading the abstracts and the reviewers agreed on 37 potentially pertinent articles, which were submitted to full-text analysis. Thirty were excluded. The reasons for exclusion are shown in Table 1. Finally, seven SR (with two meta-analyses) were assessed (Fig. 1).

### Studies characteristics

SRs were carried out in 5 countries: one in USA [44], two in Sweden [15, 45], one in Brazil [46], three in Spain [47, 48], and one study was a carried out in USA, Spain, and China [49]. All reviews searched PubMed/MEDLINE [15, 44–49], two Embase [46, 48], two Cochrane Oral Health Group Trials Register [15, 48], one Cochrane Central register of Controlled trials [48], one Cochrane Library databases [46], and one searched Web of Science [15]. Three SRs did not report number of patients and/or ZIs [44, 46, 47]. Five SRs showed reported explicit adherence to PRISMA guidelines [15, 45, 46, 48, 49]. The searches on databases were performed from March 2012 [45] to June 2016 [44]. Only one of the reviews [48] used a modified criteria according to the PRISMA 2009 checklist statement risk of bias of the primary studies [17], but the rest did not specify a distinct tool for risk of bias assessment [15, 44–47, 49]. Table 2 shows the characteristics of included reviews.

**Table 2:** Risk of bias assessment of included SRs according to AMSTAR risk of bias assessment tool

Systematic review (first author, year)	AMSTAR domains											
	1	2	3	4	5	6	7	8	9	10	11	Total score (quality)
Wang et al. (2015) [49]	Y	Y	Y	Y	N	Y	N	N	Y	Y	N	7 (medium quality)
Tuminelli et al. (2017) [44]	N	Y	N	N	N	Y	N	N	NA	NA	N	2 (low quality)
Molinero-Mourel et al. (2016) [47]	N	Y	N	Y	N	Y	N	N	N	N	N	3 (low quality)
Goiato et al. (2014) [46]	Y	Y	Y	N	N	Y	N	N	Y	N	N	5 (medium quality)
Chrcanovic et al. (2013) [45]	Y	Y	N	Y	N	Y	N	N	Y	N	N	5 (medium quality)
Chrcanovic et al. (2013) [15]	Y	Y	Y	Y	N	Y	N	N	Y	N	N	6 (medium quality)
Aboul-Hosn Centenero et al. (2018) [48]	Y	Y	Y	Y	N	Y	Y	Y	Y	Y	N	9 (high quality)

*Y* yes (one point), *N* no (0 point), *NA* not applicable (0 point), *CA* can’t answer (0 point)

### Quality assessment

The quality of the included SR is shown in Table 3. According to AMSTAR risk of bias assessment tool, SRs were mostly categorized as medium quality (four SRS [15, 45, 46, 49]), two were of low quality [44, 47], and only one review was high quality [48]. The mean score (±SD) of included reviews was 5.28 (±2.36). The minimum and maximum scores were 2 and 9 respectively: (i) All SRs were assessed by two reviewers and scored yes; (ii) none of reviews reported potential sources of support and conflict of interest for systematic review and/or each of included studies; (iii) none of them reported reason for exclusion; (iv) only one review reported a specific tool for quality assessment [48].

**Table 3:** Results of risk of bias assessment by AMSTAR tool

Author (year), country of origin, funding(Y/N/?)-Conflict of interest(Y/N/?) [JCR]	Research question or objectives	Primary outcome(s)	Number and designs of included studies (number of patients and implants)	Control group	SR/SR and M	Databases searched (search date)	Follow-up	Quality assessment tools	Implant loading (immediate/late)	Type of edentulism (total/partial)	Main findings	Main conclusion
Wang et al. (2015) [49], China and Spain, (Y-N) [Q1; 1,96]	To assess the predictability of oral rehabilitation by four zygomatic implants with no anterior support in regard to implant survival, technical and biologic complications	The survival rate of the zygomatic implants	3 human clinical trials (2 prospective and 1 retrospective) (49 patients, 196 ZIs)	None	SR and M	(from September 2000 to November 2013)	30–363 (mo)	None	Immediate loading	Fully edentulous	- Zygomatic implant survival rate weighted mean (WM) was 96.7% (range, 95.8 to 99.9%), with a 95% confidence interval (CI) of 92.5 to 98.5%. - Limited number of surgical complications and prosthetic complications	Maxillary rehabilitation by four zygomatic implants with no anterior support is a reliable approach
Tuminelli et al. (2017) [44], USA, (NR-NR) [2.39]	To systematically review the outcome of immediate loaded zygomatic implants	Immediate load survival, complications	38 articles	None	SR	PubMed (from 1990 until June 2016)	At least 12 months follow-up (according to text follow-ups ranged between 1 and 10 (y))	None	Immediate loading	Both totally or partially edentulous maxilla	- The success of implants and prostheses ranged from 96 to 100%. - The complication rates are relatively few, rarely catastrophic, and easily managed	Immediately loading zygomatic implants for the restoration of the severely atrophic maxilla presents a viable alternative for treatment of the atrophic maxilla and is recommended for the restoration of the severely atrophic maxilla with or without anterior conventional implants
Molinero-Mourel et al. (2016) [47], Spain, (NR-N) [1.07]	To analyze and describe the most frequent surgical complications associated with the use of zygomatic implants	Complications	13 articles (1 SR, 5 pros, 5 retros, 1 pros and retros, 1 cohort) (3240 ZIs)	None	SR	PubMed (up to December 2015)	1–12 (y)	None	All studies were immediate loading	Both totally or partially edentulous maxilla	- Out of the most frequent surgical complications, sinusitis (3.9%) and failure in osseointegration (2.44%) are highlighted	Rehabilitation using zygomatic implants is a consolidated therapeutic option although it does not lack in possible complications; therefore, it should be reserved only to professional clinicians with vast surgical experience
Goiato et al. (2014) [46], Brazil (N-N) [1.52]	To evaluate clinical studies on the follow-up survival of implants inserted in the zygomatic bone for maxillary rehabilitation.	Survival of implants	25 articles (design NR) (748 ZIs)	None	SR	PubMed/MEDLINE, Embase, and Cochrane Library databases (from 2000 to July 2012)	Mean follow-up: 42.2 (mo) (range 0–144)	None	Fifteen studies conducted late loading (prosthesis insertion at 4–6 months after initial implant loading), whereas 10 studies reported immediate loading	Both totally or partially edentulous maxilla	- These studies reported the insertion of a total of 1541 zygomatic implants and 33 implant failures -After a 36-month follow-up, the survival rate was 97.86%	- The survival of osseointegrated implants may also be related to the use of suitable presurgical examinations and the parameters used during the surgical procedures - Zygomatic implants appear to be an effective alternative for the treatment of an atrophic maxilla.
Chrcanovic et al. (2013) [45], Sweden (NR,N) [1.66]	“What is the survival rate of zygomatic implants (ZIs)?” and “What are the most common complications related to surgery of zygomatic implants?”	Survival rate, complication	42 article, 1145 patients and 2402 ZIs	None	SR	PubMed (Up to March 2012)	Range: 6–120 (mo)	None	Between 42 studies, 12 evaluated the use of ZIs applied with immediate function protocols	Both totally or partially edentulous maxilla	- 12 evaluated the use of ZIs applied immediate protocol and 3 after maxillary resections for tumor ablations (showed lower success.). - Of 2402 ZIs, 56 ZIs were reported as failures - The CSR over a 12-year period was 96.7%.	- ZI placement needs very experienced surgeons since delicate anatomic structures such as the orbita and brain may be involved - Despite the high survival rate observed, there is an impending need for further investigations
Chrcanovic et al. (2016) [15], Sweden (NR-N) [1.66]	To assess the survival rate of zygomatic implants (ZIs) and the prevalence of complications based on previously published studies	Complications	Sixty-eight studies were included one randomized clinical trial,16 prospective studies, and 51 retrospective analyses, comprising 4556 ZIs in 2161	Conventional implants	SR	PubMed/Medline, Web of Science, and the Cochrane Oral Health Group Trials Register (up to December 2015)	Range: 1–144 (mo)	None	26 studies immediate loading and studies 34 studies evaluating delayed loading protocols	Both totally or partially edentulous maxilla	Immediate loading showed a statistically lower ZI failure rate than other studies (*P* = .003). - Studies evaluating ZIs for the rehabilitation of patients after maxillary resections presented lower survival rates. - Postoperative complications: sinusitis, 2.4% soft tissue infection, 2.0%, paresthesia, 1.0% and oroantral fistulas, 0.4%	- ZIs present a high 12-year CSR, with most failures occurring at the early stages postoperatively. - Main complication was sinusitis, which can appear several years after placement
Aboul-Hosn Centenero et al. (2018) [48], Spain (Y-N) [1.15]	To compare the survival rates (SRs) of oral rehabilitations performed with 2 zygomatic implants (ZIs) combined with regular implants (RIs) versus 4 ZIs	Survival rates	6 articles (4 prospective and 2 retrospective case series) A total of 130 ZIs and 186 conventional implants were placed in 64 patients.	4 ZIs (case) versus2 ZIs combined with regular implants RIs (control)	SR and M	MEDLINE/PubMed, Cochrane Central register of Controlled trials Cochrane Oral Health group Trials Register, and EMBASE between 2007, and June 30, 2015	Range: 12–82 (mo)	The criteria were modified according to the PRISMA 2009 checklist statement [17]	Immediate loading	Fully edentulous	- ZIs SR weighted mean was 98.0%, CI [96.7 to 99.8%]. For the control group (2 ZIs + 2 RIs) and the test group (4 ZIs), the implant SR was 98.6% and 97.4%, respectively - No statistically significant differences in terms of SRs were obtained between both groups, *P* = 0.286.	- No statistical differences in 2 groups in terms of survival and failure rates. The reduction on treatment time and morbidity related to regenerative approaches may be its main advantage. - The zygoma quad seems to be the treatment of choice for the severely atrophic maxilla.

Legends: Y yes, N no, ZIs zygomatic implants, SR systematic review, M meta-analysis, Mo month(s), Yr year(s)

### Results of SR

Results of systematic reviews regarding survival rates and complications are listed below; please check the main outcomes in Table 2 for more information.

#### Survival rate/failure rate

The included SR reported promising results for ZIs placed in atrophic jaws. Six SR evaluated the survival rate of ZIs in atrophic jaws and 2 performed meta-analysis. The survival rate reported ranged from 95.2 to 100% although ZIs used for the rehabilitation of patients with resected maxilla’s established higher failure rates up to 21.43% (78.57%). Four SR assessed the survival rate of oral rehabilitation using ZIs [15, 44–46]. One SR with M assessed the survival rate of edentulous maxilla rehabilitation only by the zygoma quad (4 ZIs) [49], while a SR with M compare the survival rates of oral rehabilitations performed with zygoma quad with 2 ZIs with standard implants [48].

In the study by Chrcanovic et al. [45] in 2013, forty-two studies were assessed of those 3 reported the use of ZIs for rehabilitating patients after maxillary resections for tumor ablations. A total of 2402 ZIs (1145 patients) were reviewed and only 56 failures were reported. Nine implant failures were related to 642 ZIs applied with immediate function protocols. ZI failures were mostly reported 6 months after the surgery of implant placement (the abutment connection phase) or before. The CSR over a 12-year period was 96.7%.

In another SR published 3 years later, Chrcanovuc et al. [15] in their updated review reported on the publications included 2161 patients and 4556 ZIs, with a total of 103 ZI failures. The probability of an event (ZI failure) was 1.3% (95% confidence interval [CI], 1.0–1.6; standard error, 0.2; *P* < .001; heterogeneity, *t*^2^ = 0.000, c^2^ = 69.183, df = 67, *I*^2^ = 3.155%, *P* = .404). The CSR over a 12-year period was 95.21%. Of 68 studies, in 26 articles, ZIs were loaded immediately and established a higher survival rate than the delayed group (1074 patients, 2219 ZIs; 37 failures; 1.67%). This difference was prone to be statistically significant (*P* = .003 by the Pearson chi-squared test). In 5 of 68 included studies, ZIs were used for the rehabilitation of patients with resected maxilla’s with a survival rate ranging from 78.6 to 94.1%.

In a SR assessing ZIs in 25 studies reporting on a total of 1541 ZIs with 33 implant failures (The number of patients range from 4 to 76, mean = 29.9 patients), the survival rate was 97.86% after 36 months. This value remained constant up to the last follow-up period [46]. The results suggested that the survival of ZIs decreased by loading (after 12 months and 24 months for late and immediate implants respectively). No statistical test was conducted to show the significance of any relationship between the number of implants in each study and the failure rate. Only one study established a higher failure rate (21.43%) compared to the others which utilized ZIs for reconstruction of extensive maxillary defects [50]. The failures were mainly related to clinical complications and mainly happened during the first year after surgery.

Tuminelli et al. [44] assessed 38 articles with at least 12 months follow-up and stated that in the immediate loading of ZIs the success of implants and prostheses ranged from 96 to 100%.

In a SR in 2015, the survival rate of edentulous maxilla rehabilitation by zygoma quad was assessed [49]. A total of 196 ZIs were placed in 49 patients, and the weighted mean (WM) of implant survival rate was 96.7% (ranging from 95.8 to 99.9%), with a 95% CI [92.5 to 98.5%]. Of 196 ZIs, 6 ZIs failed; 2 at 6 months, 1 at 30 months, and 3 (in one patient) 7–9 months after surgery.

Likewise, a SR with meta-analysis compared the survival rate of edentulous maxilla rehabilitation by means of 2 ZIs combined (with regular implants) with 4 ZIs (zygoma quad) [48]. Meta-analysis was performed using six articles. A total of 512 implants (326 ZIs and 186 regular implants) were placed in 64 patients. Total weighted mean was 98.0%, 95% (CI) of 96.7 to 99.8% (98.6% and 97.4% for each group respectively). This survival rate tended to increase over time. There was no statistically significant difference between the survival rates of the two groups (*P* = 0.286).

#### Complications

Six SR reported on surgical and prosthetic complications when placing ZIs [15, 44–47, 49]. Sinusitis was the most frequently reported complication in all included reviews but the most important complications reported were orbital cavity penetration [49] and orbital paresthesia [45]. In the included reviews, there were studies with no complications. Likewise, some included studies in reviews did not mention the presence or absence of any complication; therefore, the numbers reported may be underestimated.

Concerning the complications with the ZIs or the ZI surgery, Chrcanovic et al. [45] assessing a total of 2402 ZIs in 1145 patients, reported 48 cases of peri-implant soft tissue infection,70 cases of sinusitis, 15 of nerve paresthesia, and 17 cases of oroantral fistulas. Three years later in an updated review, they reported 127 cases of sinusitis (total 3707 ZIs, 2.4%, 95% confidence interval CI [1.8–3.0]), 67 events of gingival infection around the implants (total 2190 ZIs, 2.0% 95% CI [1.2–2.8]), 28 events of paresthesia (1.0%, 95% CI [0.5–1.4]), and 25 episodes of formation of oroantral fistulas (0.4%, 95% CI [0.1–0.6]) [15].

In the study by Molinero-Mourele et al. [47] in 2016, sinusitis was considered the most frequently observed complication, with an average prevalence of 3.9 ZIs out of every 100 placed. Likewise, the prevalence of local infections or mucositis was 4%. Bruising ranks fourth place in terms of frequency, with 3.9%. Non-osseointegrated implants appear also with a mean frequency of 2.44%, while the frequency of oroantral communication was reported as 2%. They also stated labial laceration as one of the most common complications, but one study reported it [51].

In the study by Tuminelli et al. [46], regarding sinusitis, 26/332 patients reported sinusitis, 8 studies had no case of sinusitis, and others had not reported the number of sinusitis. Regarding prosthetic complications, some studies reported cases of loosening screw, cases of fractured screw, prosthesis, denture, framework, and fractured anterior teeth. The number of complications might be underestimated as some studies did not mention complications. The complications were few, rarely catastrophic and easy to manage.

Wang et al. [49] noted few occurrences of surgical/prosthetic complications. Three (6%) patients developed sinusitis. There was one (2.04%) case of infection followed by the formation of a fistula, one case of cheekbone hypoesthesia (2.04%), and some cases of soft tissue inflammation around the abutments (caused by poor oral hygiene and/or improper prosthetic design), but the most significant case was orbital cavity penetration caused by drilling during surgical placement. Regarding prosthesis complication, one abutment screw and two prostheses fractures were noted. In addition, one implant had an unfavorable position and was considered as “sleeping.” No other complications were reported.

In another study, 1541 ZIs were assessed [46]. Sinusitis was the most frequently reported complication (40 cases (5.34%)). Clinical complications reported before final prosthesis insertion were as follows: 24 (3.20%) cases of sinusitis (13%) OA communication, 1 (0.13%) bucosinus fistula, and 1 (0.13%) chronic gingivitis. Regarding clinical complications after FP insertion, 16 cases of sinusitis (2.13%), one case of OA communication, 1 fixed denture loss (0.13%) (due to 3 ZI loss), and also one case of paresthesia of the infraorbital nerve were reported. Prosthetic complications were artificial teeth fracture, removal of a fixed denture or overdenture, and allergy to gold alloy of the metal framework. Other complications reported were infraorbital swelling and discomfort or pain symptoms in the zygoma, facial edema, redness, gingivitis, poor oral hygiene, moderate nasal bleeding, facial hematoma, lip burning, etc. One SR reported no information regarding complications [48].

## Discussion

Considering the growing number of SR on this clinically important topic, the important next step is to provide an overview of current SR with a high level weight of evidence to guide decision makers in healthcare. An umbrella review is an approach to analyze published SR in order to compare and contrast the findings of SR on a defined topic [52]. To the authors’ best knowledge, this is the first overview to summarize findings and conduct a critical appraisal and risk of bias assessment of SR evaluating clinical variables of ZIs placed in atrophic maxilla (survival/failure rate, surgical, and prosthetic complications).

Our results suggested that in the severely atrophic maxillae, ZIs can be a favorable choice as they showed good/excellent survival rates ranging from 95.2 to 100% even in long-term follow-ups (> 10 years), and this is comparable to conventional implants. Oral rehabilitation using implant placement has always been more sensitive in the maxilla than mandible. Furthermore, this gets more challenging when the available bone becomes insufficient and limited due to bone atrophy or maxillary sinus pneumatization, or a combination of both [35]. To solve this problem, in the severely atrophic maxilla, different protocols have been advocated; different grafting techniques [53], sinus floor elevation [54], GBR (guided bone regeneration), and ZIs [44]. The known gold standard procedure for oral rehabilitation was the reconstruction of the resorbed alveolae using autogenous bone grafts. They are supported by a notable body of scientific evidence [4]. Different types of grafts are available for ridge augmentation. In severe atrophy, large quantities of bone are needed to be able to place conventional endosseous dental implants, and for large regenerations, onlay grafts are the preferred method [55]. Bony defects are reconstructed either in conjunction with dental implant placement or as a separate surgery procedure prior to dental implant placement (staged approach). The latter is preferred in large defects where primary stability cannot be achieved properly [4]. Although being widely used, bone grafting techniques suffer a series of shortcomings, both clinical and biological, that prevents high success rates as of alternative treatment based on ZIs; they are associated with longer treatment times as it takes a minimum of 4–6 months for bone formation in regenerated sites to be able to place dental implants [56]. Moreover, research suggests that implants placed in pristine bone experience higher survival rates than in grafted bone [55, 57].

In 1998, Branemark et al. developed a technology for oral rehabilitation in severely atrophic maxilla named “zygomatic technique” in which zygoma bone was utilized for implant anchorage. ZIs were defined as machined surfaced self-tapping implants in commercially pure titanium that are available in different lengths ranging from 30 to 52.5 mm used for oral rehabilitation without the use of grafts [35]. They suffered various technical modifications over time. Compared with major bone grafting techniques, ZI placement is less invasive with long-term high survival rates and can be a good alternative to grafting techniques esp. in patients in whom bone grafts cannot be harvested [49]. Even when the crestal bone of the maxilla is not sufficient, ZIs have a minimum of 8–10 mm implant-bone contact. The engagement of as much cortical bone as possible has been used as it is a decisive factor for primary stability and therefore higher success rates [27]. ZIs have the anchorage in four cortical portions (the palatina alveolar crest and sinus floor are extra cortical portions used) compared to one or a maximum of two cortical portions with regular implant placement [15, 58]. However, this is not always the case as according to ZAGA classification suggested by Aparicio et al. [59] the extra-maxillary approach many times is only anchored in the zygoma. The critical success factors of ZI-based therapy are the primary stability as well as establishing a harmonious occlusion (book). Our findings are in line with Apricio et al. who established that in many cases, ZIs had shown better clinical results compared with bone grafting procedures representing a possible new “gold standard” for atrophic jaw treatment [11].

### ZIs in resected maxillas

In cases with total/partial maxillectomy, ZIs may be used for maxillary reconstruction as an alternative to other treatment options like non-implant retained obturators and microvascular free flap ZIs. As detailed, the frequency of failure rates varied between 0 and 4.8% except for ZIs used for the rehabilitation of patients with resected maxillas that the number reached 21.43% (78.57% survival rate). In some patients, recurrent infections, recurrent tumors, a small volume of available bone (for anchorage and osseointegration), and overloading leverage in extensive maxillectomies might improve the risk of failure. Overgrowth of soft tissues around ZIs might create deep pockets which make such patients more susceptible to peri-implantitis [15, 60]. Also in patients treated with radiotherapy, deleterious effects of radiotherapy on bone reparative capacity have been reported which negatively affects the survival rate of ZIs [61]. Therefore, to lower failure rates of ZI placement in resected maxilla, implant placement in medically compromised patients and patients with a history of previous head and neck radiation as well as general contraindications of implant surgery are now counted as a contraindication of ZI placement [35, 62].

### Zygoma quad/2 ZIs with standard implants

There was no statistical difference for survival rate of 4 ZIs with no additional anterior implant support and 2 ZIs with dental implants, and in fact, both techniques of using ZI implants suggested high survival rates (97.4% and 98.6% respectively). Generally, the combined option was preferred due to lower costs and lower risk of serious complications like orbital penetration during surgical placement [63]. In fact, the zygoma quad was the choice in patients with severe anterior maxilla bone deficiency cases in which there is not enough bone in the anterior maxillary region to allow the placement of at least two short implants [35] and required additional advanced bone grafting procedures in the anterior region to place standard implants [48, 49].

### Immediate/delayed loading

Considering the differences between immediate and delayed loading, there was statistically significant lower survival rates reported for a two-stage approach [49]. The delayed approach with multiple connections and disconnections of the transepithelial components of ZIs might disrupt the reestablishment of the peri-implant soft tissue seal, which subsequently leads to an increased risk of developing oroantral communication and the risk of sinusitis. Likewise, immediate loading was known as a primary treatment option because it reestablishes the esthetics and function without waiting for the conventional healing time necessary when using delayed protocols [6, 64, 65]. Chrcanovic et al. [15] stated that the difference in survival rate of delayed and immediate loaded ZIs might be related to longer follow-ups in delayed loading protocols, as longer follow-ups may lead to higher failure rates.

Immediate loading seems to be the choice as it allows early function and restoration. Moreover, it reduces postsurgical pain and discomfort dramatically since pain and discomfort without a temporary removable denture rubbing over a non-attached mucoperiosteal flap are much less [66].

### ZI shortcomings

Although presenting high success rates and patient acceptance, ZIs remained a relatively under-utilized treatment choice for atrophic maxilla treatment due to the following shortcomings:

1. In the original technique, it was proposed to place ZIs using an “intrasinus” approach but the major shortcoming with this approach was a greater palatal inclination than natural dentition. Palatally placed ZI heads lead to a bulky prosthesis which limits tongue space and adversely affected the speech articulation and causing a mild deterioration in speech [67]. To solve the problem, the “extrasinus” technique was advocated which provided more space for the tongue and enabled a more crestal emergence of ZIs [49, 68].
2. ZI placement is a surgery beyond the alveolar area involving placement of long tilted implants; therefore, ZI placement is a difficult surgical procedure among clinicians and its determination is dependent upon experience. There are various technical difficulties with ZIs that might lead to wrong angulation of ZI placement and the subsequent complications.
  i. Placing the apical portion of ZIs at the proper position in zygoma after sequential drilling is difficult and challenging, and a wrong angulation of drilling (tilted backward) can easily lead to infratemporal fossa.
  ii. No specific anatomical reference is determined for exit point (entry point at the zygoma bone) during surgical placement.
  iii. Limitations of mouth opening and the presence of opposing dentition increase the difficulty of surgery.
  iv. Due to anatomical variations in zygoma bone, the surgical plan should be customized for each patient accordingly (reference to book).

With regard to technical struggles as well as complexity and highly varied morphology of zygomatic bone, many may be overcome with a more thorough knowledge of underlying anatomy through digital technologies. Placing surgical guides and calibrating a navigation system can improve ZI placement accuracy and safety by minimizing adverse surgical events due to human errors [33]. However, currently guided surgery with ZIs faced limitations like inadequate surgical access for the long twist drills (inadequate mouth opening); therefore, there is no effective implant software provided for ZI fully validation. Only surgical guides for pilot drilling are currently provided and the rest of the procedure is free-hand. Considering present shortcomings with guided surgery for ZIs, real-time navigation is a good alternative. The primary results with this approach are promising [49]. Given the possibility afforded by new technologies like navigation and guided surgery, future improvements in ZI placement is near to bloom.

### Complications

By reviewing the potential complications that may occur using the ZIs published in SRs, we found a number of surgical and prosthetic complications with sinusitis being the most frequent complication reported. The complications were mainly described as mild, easy to manage, and rarely catastrophic and they rarely occur. These numbers suggest that the technique has good clinical outcomes with high predictability. Nevertheless, a few cases of serious complications like temporal injuries of the infraorbital nerve (infraorbital nerve paresthesia) and penetration of the orbital cavity while placing ZIs have been reported in the literature. Generally, the complications of ZIs can be divided into immediate and late complications; hematoma, paresthesia, pain, and orbital penetration are examples of immediate complications. Immediate complications mainly have a good prognosis. Loss of osseointegration, oroantral communication, chronic sinusitis, and soft tissue infections are counted as late complications which need careful treatment and considering the anatomical site [21].

### Sinus infection (sinusitis)

Maxillary sinus infections reported in the literature regarding ZI placement can be bilateral or unilateral. After all maxillary sinus-related surgeries, the sinus cavity fills up with blood and becomes radiopaque for a while after the surgical procedure [69]. Some have suggested that in the original protocol, ZIs traversing the maxillary sinus and engaging the palatal bone in the coronal aspect had a higher incidence of sinusitis than in the new techniques in which implants are placed in a more lateral and vertical position, negating the need to introduce a foreign object into the sinus, therefore reducing the risk of developing postsurgical sinusitis [44, 70].

However, some have shown that ZIs protrude or transverse the sinus cavity which may cause sinus membrane thickening without the clinical signs of sinusitis [71] which implies that ZIs do not act as a foreign body causing chronic sinusitis when penetrating the sinus [24]. Moreover, lack of osseointegration at the palatal margin may result in transversal mobility of the ZIs causing a bacterial pump effect during function [72]. In fact, bacterial migration from the oral cavity might be one of the reasons of sinusitis [15]. Another reason reported was postsurgical debris left inside the sinus cavity; it can migrate and cause physiologic blockage of the maxillary ostium (OMC) which may result in recurrent sinusitis resistant to chemotherapy and needs surgery [73]. Sinus infection was among the most common complications reported but seems not to impair osseointegration [15]. The new techniques advocated for ZI placement (for instance, sinus slot technique) have proved to reduce the rate of postsurgical sinusitis by avoiding sinus penetration [64]. The clinical management and treatment of patients with signs and/or symptoms of postsurgical sinus infection includes antibiotic therapy considering Augmentin, Amoxicillin, Bactrim/Septra DS, Zinacef, or Levaquin. Systemic decongestants may be prescribed to ease sinus drainage. In cases of OMC with recurrent sinusitis resistant to chemotherapy, drainage of the infected maxillary and ethmoid sinuses should be considered [24, 73].

### Oroantral communication

Oroantral communication is a communication between oral cavity and maxillary sinus, caused by the weak sealing between the bone and implant head. Some reasons have been stated:

1. Fracturing the thin alveolar crest as well as over-countersinking during implant installation [51]
2. The hole in the machined Branemark system which is designed for the abutment screw seems to cause oroantral communication [74].
3. Delayed loading of ZIs has multiple connections and disconnections of the transepithelial implant components which slows the establishment of peri-implant soft tissue barrier causing oroantral communication [64].

#### Fractured implant

Regarding prosthetic complications, there were reports of fractured ZIs (fractured screw, abutment, and prosthesis) [44, 49]. Also, the “loss” of arch splinting is mainly reported as a consequent result of fractured implant. To minimize such problems, splinting cross arch is highly recommended as unsplinted ZIs face significant stress due to occlusal forces at implant platform as well as superstructure prosthesis in centric and lateral loadings. A periodic follow-up of quarterly or biannual appointments to check stability of abutment screws/prosthesis as well as peri-implant soft tissues is warranted. To remove a fractured ZI, it should be noted that the apical part of the implant is osseointegrated, and therefore with the understanding that these implants are osseointegrated, for disengaging it, extreme caution in turning the implant in a counterclockwise direction should be advocated [24].

#### Penetration of orbital cavity

The improper angle of entrance of the starting drills may result in a potentially wrong medial trajectory of drilling which may drill “the body of zygoma.” To avoid such serious complications, the clinicians should take extensive care of anatomical landmarks when placing ZIs [47].

In the SR by Wang et al. [68], only one patient experienced it in one study [36]. In the zygoma quad approach, ZIs placed anteriorly cause higher risks of orbital penetration as they might involve the wall of the orbital cavity [63].

#### Infraorbital nerve damage (paresthesia)

This complication is closely linked to surgeon expertise and the surgical team’s discipline as the nerve could be damaged by improper manipulation of the surgical flap including nerve damage during dissection and releasing the flap, excessive stretch applied to the flap, and “crushing” injuries caused by improper position of the retractor [24]. In a number of SRs, 44 cases of paresthesia were described [15, 45, 46].

#### Mucositis and peri-implantitis

Gingival infection and mucositis around ZIs are described by Molinero-Mourelle et al. [47] and Chrcanovic et al. [15] and found directly relevant to sinusitis, favored by superficial infection and lack of cicatrisation of soft tissue around the implant as well as failure in bone regeneration around ZI (lack of osseointegration). Likewise, prosthodontic rehabilitation plays an important role as the improper design of prosthesis causes difficulties in implementation of oral hygiene [63].

#### Postsurgical pain, bruising, soft tissue laceration, and burn

The incidence of such factors might be higher than the reported numbers. They are underdocumented in many studies as their clinical exhibitions are less alarming and are mostly self-limited during the postoperative period [47]. The said complications seems not be linked to the failure rate.

### Limitations and strengths

This review had some shortcomings. The defined inclusion criteria caused a degree of selection bias since we only included studies written in English. Likewise, data extractions were done by one reviewer although there was strict supervision by two authors. Another shortcoming was the small number of SRs and meta-analyses which could not be analyzed qualitatively. Additionally, the risk of bias assessment was performed by means of AMSTAR risk of bias assessment tool [43] and our included SRs were mainly classified as “moderate” quality (4 of 7 SRs). Also, there might be a degree of overgeneralization of results related to the nature of umbrella reviews. Due to the named limitations of the current study, any interpretation must be done cautiously. Future systematic reviews and primary studies should consider the following points:

1. RCTs with a large sample size and long-term follow-up are needed to make a clear comparison between ZIs and other treatment options in atrophic jaws.
2. SRs should follow available guidelines and criteria for reporting studies to improve their quality.
3. Improvements in surgical placement of ZIs have begun with free-hand surgery, progressing to guided surgery and recently to real-time navigational placement. Future studies should be directed at resolving barriers of guided surgeries and navigational placement to maximize accuracy and predictability of results.

## Conclusions

ZIs appear to be a consolidated therapeutic option for significantly atrophic maxilla offering a promising alternative to heavy bone grafting techniques with lower costs, fewer complications, shorter time for rehabilitation, less prosthodontic work needed, and significantly higher survival rates. They have been assessed in reviews with long-term follow-ups (more than 10 years) and showed reliable and predictable results. It does not lack in complications but they are mostly mild and facile to manage and rarely get catastrophic. To prevent serious complications, this treatment should be reserved only to professional clinicians with vast surgical experience and a good knowledge of the 3D anatomy. Nonetheless, this conclusion is based on a limited number of SRs and the majority of them presented moderate quality.

## Data Availability

The datasets used and/or analyzed during the current study are available from the corresponding author on reasonable request.

## Abbreviations

ZI: Zygomatic implant
SR: Systematic review
OA communication: Oroantral communication
